# Significance of the cribriform pattern in predicting the prognosis of lung adenocarcinoma patients: A systematic review and meta-analysis

**DOI:** 10.1371/journal.pone.0324376

**Published:** 2025-07-08

**Authors:** Jun Peng, Xianquan Zhang, Yan Huang, Changhui Liu, Shuyang Li, Jinfeng Liu

**Affiliations:** 1 Department of Thoracic Surgery, The Second Clinical School of North Sichuan Medical College, Nanchong, Sichuan, China; 2 Department of Thoracic Surgery, The Beijing Anzhen Hospital Affiliated with Capital Medical University Nanchong Hospital and Nanchong Central Hospital, Nanchong, Sichuan, China; 3 Department of Stomatology, North Sichuan Medical College, Nanchong, Sichuan, China; 4 Department of Thoracic Surgery, The First Affiliated Hospital of Hebei Medical University, Shijiazhuang, Hebei, China; West China Hospital of Sichuan University, CHINA

## Abstract

**Background:**

A number of studies have shown that various histological subtypes of lung adenocarcinoma have different clinical prognoses, but the cribriform pattern, as a unique histological subtype, plays an important role in the prognosis of patients with lung adenocarcinoma.

**Objective:**

In this meta-analysis, we evaluated the role of the cribriform pattern in the overall survival of patients with lung adenocarcinoma, which may provide valuable information for the treatment of patients with lung adenocarcinoma. This also provides an important basis for dividing the cribriform pattern into a new histological subtype and classifying it.

**Methods:**

We searched the literature from the PubMed, Embase, Cochrane and Web of Science online databases; extracted the data and characteristics of each study; and extracted and calculated hazard ratios (HRs) with 95% confidence intervals (CIs) to evaluate the impact of the cribriform pattern on the prognosis of patients with lung adenocarcinoma.

**Results:**

A total of 10 articles were included in the study according to the preset criteria, with a total of 5487 research subjects. The hazard ratio for the relationship between the cribriform pattern and the overall survival rate of patients with lung adenocarcinoma was 2.05 (95% CI: 1.76–2.39). The prognosis of patients with positive spread through air spaces in the cribriform pattern was significantly worse than that of patients with negative spread through air spaces in the cribriform pattern, with a hazard ratio of 2.58 (95% CI: 1.84–3.62). There was a significant difference in prognosis between patients with the cribriform pattern and those with low-grade and intermediate-grade lung adenocarcinoma, but there was no significant difference in prognosis between patients with the cribriform pattern and those with high-grade lung adenocarcinoma, with hazard ratios of 2.12 (95% CI: 1.12–4.00), 7.70 (95% CI: 2.15–36.20) and 0.96 (95% CI: 0.54–2.40), respectively. Therefore, the cribriform pattern should be used as a histological subtype of high-grade tumors, thus influencing the postoperative prognosis of patients with lung adenocarcinoma.

**Conclusion:**

The presence of the cribriform pattern is an independent risk factor for postoperative overall survival in patients with lung adenocarcinoma. The cribriform pattern should be considered a new histological subtype of lung adenocarcinoma and classified with solid carcinoma and micropapillary adenocarcinoma as high-grade tumors.

## Introduction

Lung cancer is the tumor with the highest mortality rate in the world [1], and lung adenocarcinoma (LUAD) is currently the most common histological subtype, accounting for 40% of all cases [2]. Lung adenocarcinomas are histologically heterogeneous [3] and are usually composed of a mixture of multiple growth modes. At present, when the World Health Organization (WHO) classifies lung cancer, most adenocarcinomas are mixed subtypes [4]. The International Association for the Study of Lung Cancer (IASLC), American Thoracic Society (ATS), and European Respiratory Society (ERS) [5] classify lung adenocarcinomas on the basis of their main patterns, including adenocarcinoma in situ, microinvasive adenocarcinoma, acinar, solid, micropapillary, papillary, and squamous pathological subtypes. Various histological subtypes of lung adenocarcinoma have different clinical prognoses [6]. There is a unique histological pattern called the cribriform pattern, which was included in lung adenocarcinoma by the World Health Organization (WHO) in 2015 [7]. The cribriform pattern is defined as invasive back-to-back fusion of tumor glands, poorly formed glandular spaces, lack of stroma, or invasive nests of tumor cells creating glandular cavities without solid components [8]. The main pathological subtypes of lung cancer are classified into low, intermediate, and high grades. The low grades include adenocarcinoma in situ, microinvasive adenocarcinoma and squamous, the intermediate grades include papillary and acinar, and the high grades include micropapillary and solid [4]. Most studies have shown that the prognosis of patients with the cribriform pattern is similar to that of patients with high-grade tumors. Therefore, we studied whether the cribriform pattern has an impact on the overall survival of patients with lung adenocarcinoma and whether the cribriform pattern should be considered a new histological subtype of lung adenocarcinoma.

## Materials and methods

### Search strategy

The studies were retrieved from the PubMed, Embase, Cochrane and Web of Science online databases by two reviewers. The following keywords were used to search for relevant studies: “cribriform” and “lung adenocarcinoma” or “pulmonary adenocarcinoma” and “prognostic”. The selected studies were published before September 2024. Two authors independently and manually screened the reference lists of the original articles for further relevant research.

### Inclusion and exclusion criteria

The inclusion criteria for studies were as follows: (1) patients with lung adenocarcinoma were included as research subjects; (2) patients underwent surgical resection for therapeutic purposes; (3) the relationship between the cribriform pattern and the overall survival rate of patients with lung adenocarcinoma was studied; and (4) they study type was a randomized controlled trial (RCT) or an observational study. The exclusion criteria for studies were as follows: (1) included tissues or materials from animals rather than from humans; (2) research subjects had a history of other malignant tumors; (3) research subjects had undergone preoperative adjuvant chemoradiotherapy; (4) survival results were not reported or could not be calculated; and (5) overviews, seminar papers, reviews, reports, letters and duplicate publications.

### Data extraction and quality assessment

Two reviewers independently screened the detected studies, and any disagreements were discussed and resolved with a third reviewer. Based on the Preferred Reporting Items for Systematic Reviews and Meta-Analyses (PRISMA) statement, we extracted the following data: first author’s name, publication year, country and region, number of study subjects, follow-up time (basic unit: months), HR, 95% CI, overall survival (OS) and recurrence-free survival (DFS) (Table 1). For some HRs and 95% CIs that were not reported in the literature, we used Engauge Digitizer 11.1 to read the relevant data from the Kaplan‒Meier curve and then estimated the HR and 95% CI. When univariate and multivariate analyses were performed simultaneously, we selected the latter as a more precise treatment of the results.

**Table 1:** Basic characteristics of the included studies.

Study ID	Patients	Stage I	Country	Year	HR	LL	UL	Prognosis	Follow up time	Grade	STAS
Ding Q, et al. [9]	208	86	China	2019	2.820	1.679	4.736	DFS	60 months		+
					2.729	1.737	4.288	OS			+
Zhang R, et al. [8]	279	85	China	2019	1.766	1.054	2.959	DFS	100 months		+
					2.974	1.518	5.830	OS			+
Moreira AL, et al. [10]	249	249	US	2014	2.524	0.330	19.316	DFS	100 months	Intermediate, High	–
Qu Y, et al. [11]	395	205	China	2015	1.820	1.218	2.719	DFS	100 months		–
					1.939	1.352	2.781	OS			–
Warth A, et al. [12]	674	264	Germany	2015	2.990	1.010	11.920	DFS	120 months		–
					1.720	0.310	11.310	OS			–
Nakajima, et al. [13]	1057	850	Japan	2021	1.550	0.334	7.183	DFS	200 months	Low, Intermediate, High	–
Kadota, et al. [14]	1038	1038	Japan	2014	1.483	0.676	3.254	DFS	160 months	Low, Intermediate, High	–
Kadota, et al. [15]	735	490	Japan	2019	2.754	0.724	10.473	DFS	100 months	Low, Intermediate, High	–
					2.298	1.249	4.230	OS			–
Bossé, et al. [16]	676	407	Canada	2022	1.200	0.759	1.900	OS	167 months	Low, Intermediate, High	–
Talvitie, et al. [17]	176	95	Finland	2020	2.588	1.500	4.460	OS	167 months		–

OS, overall survival; PFS, progression free survival; HR, hazard ratio; LL, lower limit; UL, upper limit; STAS, spread through air spaces.

Two reviewers independently assessed the quality of the selected studies using the guidelines of the Newcastle–Ottawa Quality Assessment Scale (NOS), with scores ranging from 0 to 9 for each study. The scoring method includes three parts: selection (0–4 points), comparability (0–2 points) and result evaluation (0–3 points). Studies with NOS scores ≥6 were considered to be of high quality (Table 2) .

**Table 2:** Newcastle–Ottawa quality assessment scale (NOS).

Study ID	Selection				Comparability	Exposure			Scores
	Representativeness of the exposure groups	Selection of the unexposed groups	Ascertainment of the exposure factors	Determination of the outcome indicators to be observed at the beginning of the study	Control for important factors	Assessment of outcome	Was follow up long enough for outcome to occur	Adequacy of follow up of cohorts	
Ding Q, et al.	*	*	*	*	**	*			7
Zhang R, et al.	*	*	*	*	**	*	*		8
Moreira AL, et al.	*	*	*	*	*	*	*		7
Qu Y, et al.	*	*	*	*	*	*	*	*	8
Warth A, et al.	*	*	*	*	*	*	*		7
Nakajima, et al.	*	*	*	*	*	*	*	*	8
Kadota, et al.	*	*	*	*	*	*	*		7
Kadota, et al.	*	*	*	*	*	*	*	*	8
Bossé, et al.	*	*	*	*	*	*	*		7
Talvitie, et al.	*	*	*	*	*	*	*		7

The protocol was registered on the PROSPERO website with the registration number CRD42024562637. The relevant registration information can be obtained from the following website: https://www.crd.york.ac.uk/prospero/#searchadvanced.

### Statistical analysis

STATA 14.0 was used to conduct the meta-analysis. For unknown HRs, we obtained Kaplan‒Meier curves from the original literature by using the software Engauge Digitizer. Heterogeneity was assessed via Cochran’s Q test and Higgins’ I^2^ statistic. When I^2^ < 50% or p > 0.10, the heterogeneity was considered low, and the fixed effects model was adopted. When I^2^ > 50% or p < 0.10, heterogeneity was considered obvious. We first analyzed the random effects model. Publication bias was described by funnel plots and Egger’s and Begg’s bias tests.

## Results

### Characteristics of eligible studies and quality assessment

**Fig 1:**
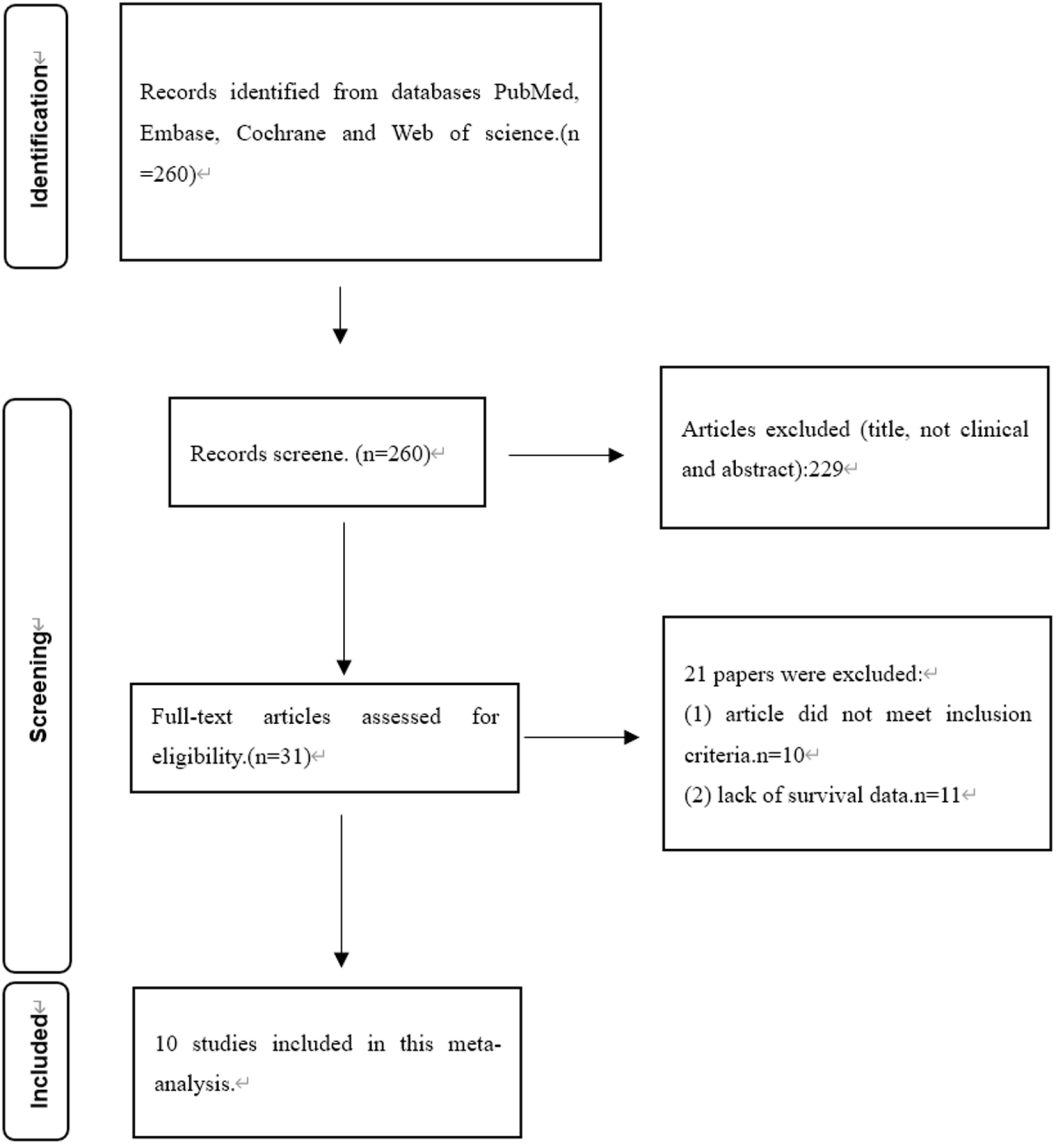
Flow diagram for retrieving eligible articles.

We searched for literature from PubMed, Embase, Cochrane, and Web of Science, and obtained a total of 260 papers. After screening for title, abstract and type of study, 229 articles were excluded based on the established criteria. We then conducted a full review of the remaining 31 articles, of which 10 were eligible for meta-analysis The results of the subgroup analysis are shown in Table 3. In a flow chart of the study selection process (Fig 1), we summarized the main features of the 10 included studies.

**Table 3:** Results of the subgroup analysis.

Subgroup	HR	LL	UL	I^2^	P	Heterogeneity between groups, p
Total	2.05	1.76	2.39	0.0%	0.573	
Grade	1.73	1.21	2.47	82.6	0.000	0.000
Low grade	4.55	2.25	9.21	63.4%	0.027	
Intermediate grade	1.93	1.37	2.73	59.1%	0.032	
High grade	0.80	0.54	1.18	66.1	0.012	
Prognosis	2.05	1.76	2.39	0.0%	0.573	0.804
OS	2.11	1.64	2.71	32.7%	0.178	
DFS	2.02	1.58	2.57	0.0%	0.840	
STAS	2.58	1.84	3.62	0.0%	0.392	
Country	2.05	1.76	2.39	0.0%	0.573	0.257
China	2.17	1.80	2.62	0.0%	0.475	
United States	2.52	0.33	19.31	0.0%		
Germany	2.50	0.91	6.93	0.0%	0.619	
Japan	1.99	1.29	3.07	0.0%	0.782	
Canada	1.20	0.76	1,90	0.0%	.	
Finland	2.59	1.50	4.46	0.0%		
Follow up time	2.05	1.76	2.39	0.0%	0.573	0.175
≤100 months	2.20	1.84	2.62	0.0%	0.790	
>100 months	1.70	1.23	2.35	8.0%	0.365	

### Meta-analysis

**Fig 2:**
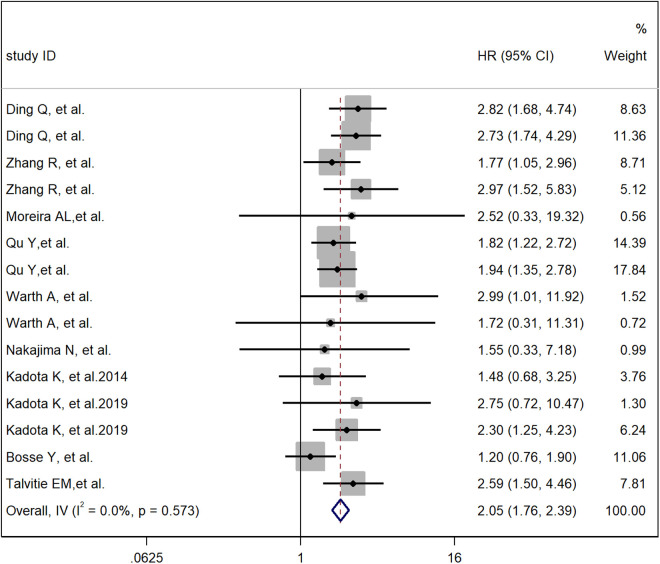
Meta-analysis of total HRs based on the cribriform pattern.

A meta-analysis method was used to study the impact of the cribriform pattern on the prognosis of lung adenocarcinoma patients. We selected a cribriform component with more than 5% identity in pathological tissues as a sieve-like pattern, and the HR of the relationship between the cribriform pattern and the overall survival rate of lung adenocarcinoma patients was 2.05 (95% CI: 1.76–2.39) (Fig 2). These findings suggest that LUAD with a cribriform component is an independent risk factor for poor prognosis. Through observation, we found that there was a significant correlation between the cribriform pattern subtype and low- and intermediate-grade tumors but no significant correlation with high-grade tumors. The pooled HRs were 2.12 (95% CI: 1.12–4.00), 7.70 (95% CI: 2.15–36.20) and 0.96 (95% CI: 0.54–2.40) (Fig 2). These findings suggest that the cribriform pattern subtype should be considered a new histological subtype of lung adenocarcinoma and classified as a high-grade tumor. In the cribriform pattern, there is a significant correlation between STAS positivity and STAS negativity in the prognosis of lung adenocarcinoma patients. Therefore, STAS positivity in the cribriform pattern is an independent risk factor for a worse prognosis in lung adenocarcinoma patients. We have analyzed all the articles in the study and analyzed the staging of lung adenocarcinoma. A total of 5487 patients were enrolled, of which 3769 were in stage I, accounting for 68.7%. This indicates that cribriform patterns are an independent factor contributing to poor prognosis in early lung adenocarcinoma patients.

### The cribriform pattern should be used as a histological subtype of high-grade tumors to influence the postoperative prognosis of patients with lung adenocarcinoma

**Fig 3:**
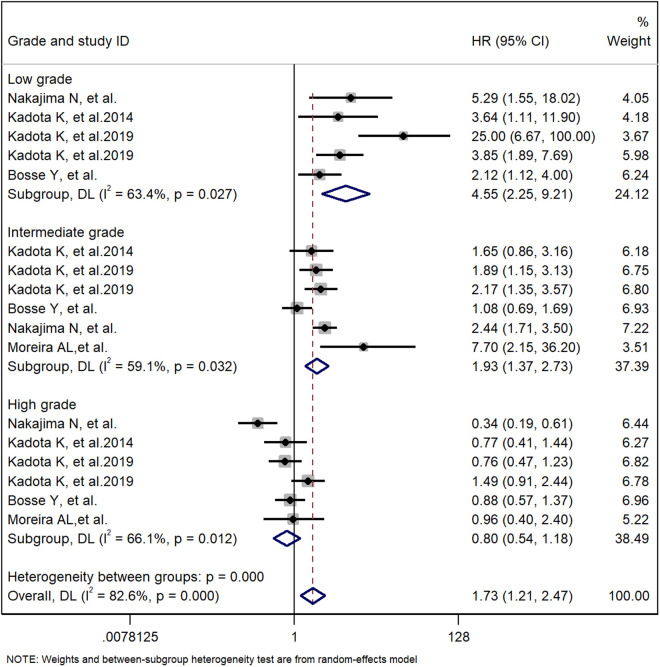
Meta-analysis of subtotal HRs based on the grade of lung adenocarcinoma.

The main pathological subtypes of lung cancer are classified into low, intermediate, and high grades. The low grades include adenocarcinoma in situ, microinvasive adenocarcinoma and squamous, the intermediate grades include papillary and acinar, and the high grades include micropapillary and solid [4]. In our meta-analysis, we compared the prognostic relationships between the cribriform pattern and low-grade, intermediate-grade, and high-grade tumors in patients with lung adenocarcinoma and obtained pooled HRs of 2.12 (95% CI: 1.12–4.00), 7.70 (95% CI: 2.15–36.20) and 0.96 (95% CI: 0.54–2.40), respectively (Fig 3). These findings indicate that there is a clear correlation between the cribriform pattern subtype and low- and intermediate-grade tumors and that the cribriform pattern subtype has a worse prognosis than lower- to intermediate-grade tumors do. There was no significant association with high-grade tumors, suggesting that both the cribriform pattern subtype and high-grade tumors have similar prognoses for patients with lung adenocarcinoma. Therefore, the cribriform pattern subtype should be regarded as a new histological subtype of lung adenocarcinoma and classified into high-grade tumors together with solid carcinoma and micropapillary adenocarcinoma.

### Patients with STAS-positive lung adenocarcinoma in the cribriform pattern have a worse prognosis

**Fig 4:**
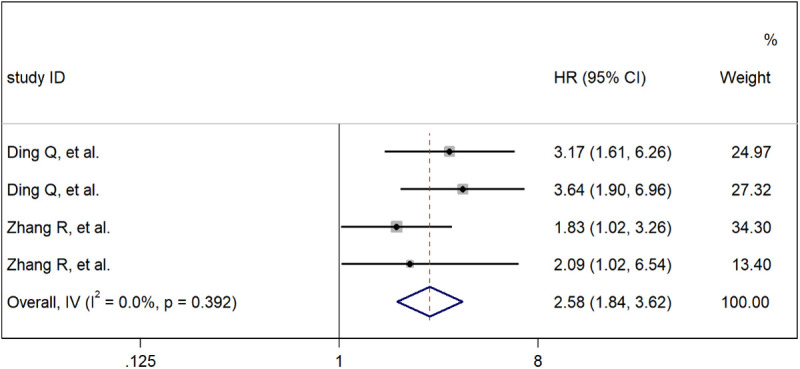
Meta-analysis of subtotal HRs based on STAS.

In our meta-analysis, we further compared the prognostic impact of STAS positivity versus that of STAS negativity in patients with lung adenocarcinoma in the cribriform pattern. Our results clearly indicate a significant correlation between STAS positivity and STAS negativity in the screening pattern and the prognosis of lung adenocarcinoma patients, with a combined HR of 2.58 (95% CI: 1.84–3.62; Fig 4). Therefore, this may indicate that STAS positivity in the cribriform pattern is an independent risk factor for a worse prognosis in patients with lung adenocarcinoma. The presence of cribriform components and a positive status should be considered synergistic prognostic factors for LUAD.

### Effect of the cribriform pattern on OS and DFS in patients with lung adenocarcinoma after surgery

**Fig 5:**
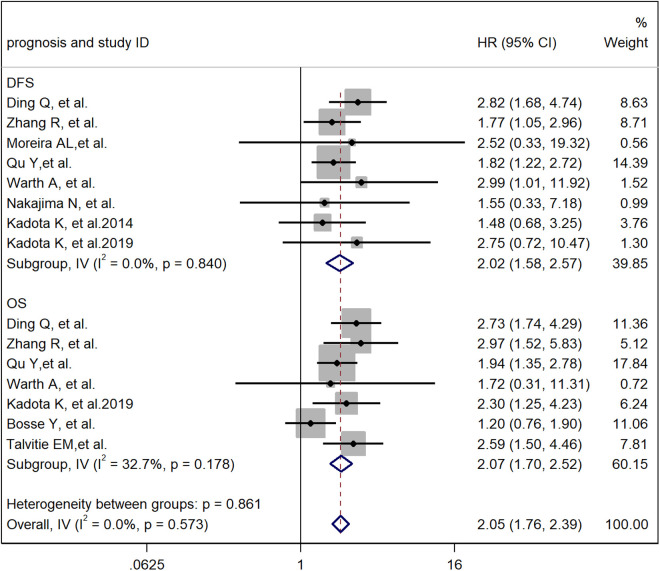
Meta-analysis of subtotal HRs based on the OS and DFS of LUAD patients.

In some studies [9], the cribriform pattern was shown to be an independent risk factor for postoperative DFS and OS in patients with lung adenocarcinoma, but other studies have denied this view [13]. In our meta-analysis, we combined these studies and compared the relationships between the cribriform pattern and DFS and OS after surgery in patients with lung adenocarcinoma. The results clearly revealed a significant correlation between the cribriform pattern and DFS, with a combined HR of 2.02 (95% CI: 1.58–2.57; Fig 5), and there was a significant correlation between the cribriform pattern and OS, with a combined HR of 2.11 (95% CI: 1.64–2.71; Fig 5). Therefore, we believe that the cribriform pattern can serve as an independent risk factor for postoperative DFS and OS in patients with lung adenocarcinoma.

### Sensitivity analysis and Publication bias

**Fig 6:**
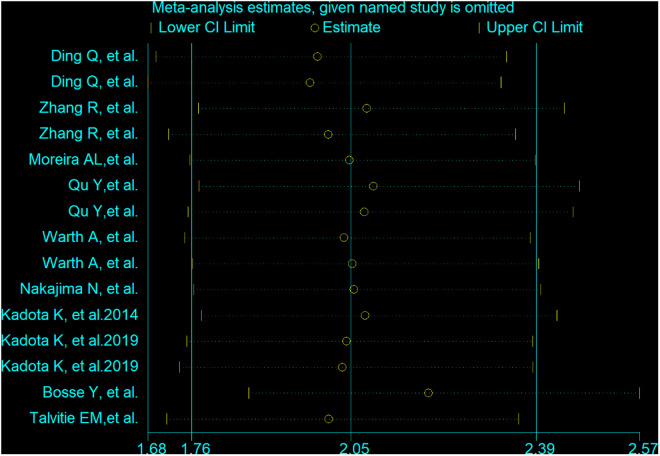
Sensitivity analysis chart.

**Fig 7:**
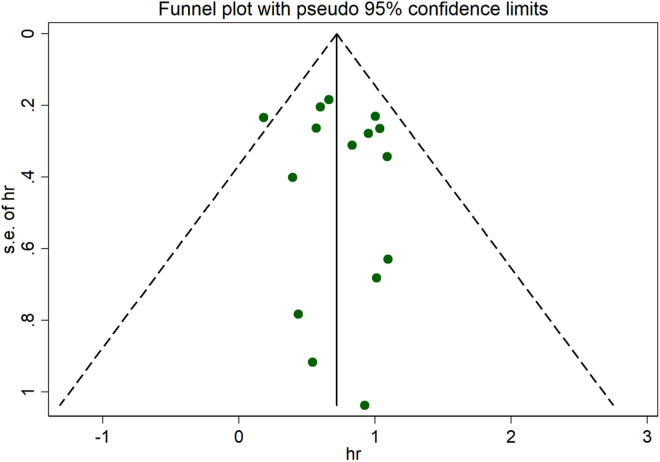
Funnel plot.

In the comprehensive meta-analysis, sensitivity analysis checks were performed to explore the sources of heterogeneity (Fig 6). Regardless of whether the fixed effects model or the random effects model was used, the results were similar. We used Begg’s funnel plot and Egger’s linear regression test to test for publication bias. Egger’s test revealed that there was no significant publication bias in this study (p > 0.05). The shape of the funnel plot was visually symmetrical, with no evidence of publication bias (Fig 7).

## Discussion

Currently, the impact of the cribriform pattern on the survival of patients with lung adenocarcinoma has been widely studied worldwide [8,18,19]. We summarized the latest progress in the research on the cribriform pattern in patients with lung adenocarcinoma in recent years, focusing on the impact of the cribriform pattern on the postoperative prognosis of patients with lung adenocarcinoma and the fact that the cribriform pattern should be regarded as a new tissue subtype and classified as a high-grade tumor type with a poor prognosis.

In this meta-analysis, we found that the cribriform pattern was closely associated with poor prognosis in patients with lung adenocarcinoma after surgery. The cribriform pattern was an independent risk factor for prognosis after surgery for lung adenocarcinoma. In addition, when the cribriform pattern was compared with low-grade, intermediate-grade, and high-grade tumors, the combined HRs were 2.12 (95% CI: 1.12–4.00), 7.70 (95% CI: 2.15–36.20), and 0.96 (95% CI: 0.54–2.40), respectively. These findings indicate that there is a significant correlation between the cribriform pattern subtype and low- and intermediate-grade tumors but that there is no significant correlation with high-grade tumors. Therefore, the cribriform pattern subtype should be considered a new histological subtype of lung adenocarcinoma and classified as a high-grade tumor along with solid carcinoma and micropapillary adenocarcinoma, which is consistent with the conclusion of the study by Q. Ding et al. [9]. In addition, we determined that there was a significant correlation between STAS positivity and STAS negativity in the cribriform pattern with respect to the prognosis of patients with lung adenocarcinoma, with a combined HR of 2.58 (95% CI: 1.84–3.62). These findings suggest that STAS positivity in the cribriform pattern is an independent risk factor for poor prognosis in patients with lung adenocarcinoma, that STAS may affect the survival of patients with adenocarcinoma with cribriform components, and that the presence of cribriform components and STAS positivity should be considered synergistic prognostic factors for LUAD. Comprehensive research clearly revealed a significant correlation between the cribriform pattern and DFS and OS, with combined HRs of 2.02 (95% CI: 1.58–2.57) and 2.11 (95% CI: 1.64–2.71), respectively. Therefore, we believe that the cribriform pattern can serve as an independent risk factor for postoperative DFS and OS in patients with lung adenocarcinoma. We can see from the figure that heterogeneity was found among the studies. We explored heterogeneity by omitting each individual study and repooling the HRs of the remaining studies, and we found that no specific study affected the overall HR. Subgroup analyses were performed, and the results presented different HRs and heterogeneity among subgroups, which may explain the source of heterogeneity in the meta-analysis. In addition, there may be no significant difference in the length of follow-up of patients (as shown in the figure). This observation may be attributed to the fact that some patients with lung adenocarcinoma have a high degree of malignancy or are already in the advanced stage when they are examined, and their survival time is relatively short; however, this is also the value of our study.

This meta-analysis is the first to investigate the effect of the cribriform pattern on the overall survival of patients after surgery for lung adenocarcinoma and to investigate whether the cribriform pattern should be considered a new histological subtype of lung adenocarcinoma and should be classified as a high-grade tumor together with solid carcinoma and micropapillary adenocarcinoma. These findings provide a prognostic basis for patients with lung adenocarcinoma with a cribriform pattern and a new treatment direction for patients with lung adenocarcinoma after surgery, providing a basis [4] for many therapeutic advances through accurate diagnosis and molecular and biomarker testing that promote molecular targeting and immunotherapy [20,21]. In addition, the cribriform pattern should be considered a new histological subtype of lung adenocarcinoma in the future and should be classified as a high-grade tumor together with solid carcinoma and micropapillary adenocarcinoma.

Although our analysis revealed that the cribriform pattern plays an important role in the prognosis of patients with lung adenocarcinoma in predicting the final outcome, several shortcomings remain. First, owing to the small number of articles and research subjects included in this meta-analysis, when more standard-design studies are performed in the future, the cribriform pattern subtype will be regarded as a new histological subtype of lung adenocarcinoma and will be classified as a high-grade tumor together with solid carcinoma and micropapillary adenocarcinoma. This conclusion will eventually be confirmed. Second, this study revealed significant heterogeneity in demographics, methods of determining the presence of the cribriform pattern in patients with lung adenocarcinoma, and measurements and adjustments for confounders. Although we used appropriate meta-analysis techniques with a random effects model, we were unable to explain this difference, but sensitivity analyses revealed that the risk estimates were reliable across various quality factors. Third, some HRs could not be obtained directly from the articles. We estimated HRs from Kaplan‒Meier curves via Engauge Digitizer version 11.1, which may reduce the reliability of our results.

## Conclusion

In conclusion, this meta-analysis demonstrated that LUAD with a cribriform component was an independent risk factor for poor prognosis. The cribriform pattern subtype should be considered a new histological subtype of lung adenocarcinoma and classified as a high-grade tumor. A positive STAS status in the cribriform pattern is an independent risk factor for poor prognosis in patients with lung adenocarcinoma, and the presence of cribriform components and positive STAS status should be considered synergistic prognostic factors for LUAD. The cribriform pattern can serve as an independent risk factor for postoperative DFS and OS in patients with lung adenocarcinoma. The above conclusions need additional research to enhance their persuasiveness.

## Supporting information

S1 File PRISMA checklist. (DOCX)

S2 File All retrieved articles. (XLSX)
